# Maternal Immunoreactivity to Herpes Simplex Virus 2 and Risk of Autism Spectrum Disorder in Male Offspring

**DOI:** 10.1128/mSphere.00016-17

**Published:** 2017-02-22

**Authors:** Milada Mahic, Siri Mjaaland, Hege Marie Bøvelstad, Nina Gunnes, Ezra Susser, Michaeline Bresnahan, Anne-Siri Øyen, Bruce Levin, Xiaoyu Che, Deborah Hirtz, Ted Reichborn-Kjennerud, Synnve Schjølberg, Christine Roth, Per Magnus, Camilla Stoltenberg, Pål Surén, Mady Hornig, W. Ian Lipkin

**Affiliations:** a Center for Infection and Immunity, Mailman School of Public Health, Columbia University, New York, New York, USA; b Norwegian Institute of Public Health, Oslo, Norway; c Department of Epidemiology, Mailman School of Public Health, Columbia University, New York, New York, USA; d New York State Psychiatric Institute, Columbia University, New York, New York, USA; e Department of Biostatistics, Mailman School of Public Health, Columbia University, New York, New York, USA; f National Institute of Neurological Disorders and Stroke, National Institutes of Health, Bethesda, Maryland, USA; g Institute of Clinical Medicine, University of Oslo, Oslo, Norway; h Nic Waals Institute, Lovisenberg Hospital, Oslo, Norway; i Department of Global Public Health and Primary Care, University of Bergen, Bergen, Norway; University of Michigan

**Keywords:** autism, birth cohort, herpes simplex virus, infection, prenatal, serology

## Abstract

Maternal infections during pregnancy are associated with risk of neurodevelopmental disorders, including autism spectrum disorders (ASDs). Proposed pathogenetic mechanisms include fetal infection, placental inflammation, and maternal cytokines or antibodies that cross the placenta. The Autism Birth Cohort comprises mothers, fathers, and offspring recruited in Norway in 1999 to 2008. Through questionnaire screening, referrals, and linkages to a national patient registry, 442 mothers of children with ASD were identified, and 464 frequency-matched controls were selected. Immunoglobulin G (IgG) antibodies to *Toxoplasma gondii*, rubella virus, cytomegalovirus (CMV), herpes simplex virus 1 (HSV-1), and HSV-2 in plasma collected at midpregnancy and after delivery were measured by multiplexed immunoassays. High levels of HSV-2 IgG antibodies in maternal midpregnancy plasma were associated with increased risk of ASD in male offspring (an increase in HSV-2 IgG levels from 240 to 640 arbitrary units/ml was associated with an odds ratio of 2.07; 95% confidence interval, 1.06 to 4.06; *P* = 0.03) when adjusted for parity and child’s birth year. No association was found between ASD and the presence of IgG antibodies to *Toxoplasma gondii*, rubella virus, CMV, or HSV-1. Additional studies are needed to test for replicability of risk and specificity of the sex effect and to examine risk associated with other infections.

**IMPORTANCE** The cause (or causes) of most cases of autism spectrum disorder is unknown. Evidence from epidemiological studies and work in animal models of neurodevelopmental disorders suggest that both genetic and environmental factors may be implicated. The latter include gestational infection and immune activation. In our cohort, high levels of antibodies to herpes simplex virus 2 at midpregnancy were associated with an elevated risk of autism spectrum disorder in male offspring. These findings provide support for the hypothesis that gestational infection may contribute to the pathogenesis of autism spectrum disorder and have the potential to drive new efforts to monitor women more closely for cryptic gestational infection and to implement suppressive therapy during pregnancy.

## Abstract (precis)

The cause (or causes) of most cases of autism spectrum disorder is unknown. Evidence from epidemiological studies and work in animal models of neurodevelopmental disorders suggest that both genetic and environmental factors may be implicated. The latter include gestational infection and immune activation. In our cohort, high levels of antibodies to herpes simplex virus 2 at midpregnancy were associated with an elevated risk of autism spectrum disorder in male offspring. These findings provide support for the hypothesis that gestational infection may contribute to the pathogenesis of autism spectrum disorder and have the potential to drive new efforts to monitor women more closely for cryptic gestational infection and to implement suppressive therapy during pregnancy.

## INTRODUCTION

Autism spectrum disorders (ASDs) comprise a spectrum of neurodevelopmental syndromes with various degrees of social impairment, deficits in language and communication, and stereotypic and repetitive behaviors (1). There are no definitive biomarkers for ASD; hence, diagnosis relies on clinical criteria (2). The prevalence of ASD is estimated as 1 to 2% in high-income countries in Asia, Europe, and North America and is higher in males than females (3, 4). The pathogenesis is in most instances unexplained; however, both genetic and environmental factors are implicated (5, 6).

Infections during pregnancy are suggested to be a risk factor in several neurodevelopmental disorders, including ASD (7–10). Mechanisms proposed for damage include placental inflammation, proinflammatory cytokine production by mother or fetus, and maternal autoantibodies that bind to fetal brain (11). Animal models of neurodevelopmental disturbances reminiscent of ASD based on prenatal exposure to viral [e.g., double-stranded RNA and poly(I)-poly(C)] and bacterial (e.g., lipopolysaccharide) mimics indicate that more general, nonspecific immune mechanisms may be operative (12–14).

Maternal infection during pregnancy with *Toxoplasma gondii*, rubella virus, cytomegalovirus (CMV), and herpes simplex viruses 1 (HSV-1) and 2 (HSV-2) (ToRCH agents) may lead to spontaneous abortion or cause intrauterine growth restriction, preterm labor, severe brain damage, or visual impairment. The risk of transmission to the fetus and disease severity depend on gestational age at the time of infection (15, 16).

Maternal rubella virus infection is not associated with fetal damage if acquired before pregnancy; however, preconceptional infection with *T. gondii* may have neurological consequences (17–20). Although primary infections during pregnancy are rare, herpesviruses, including HSV-1, HSV-2, and CMV, frequently cause persistent or latent infections (21, 22). Accordingly, maternal infection prior to pregnancy may be followed by reactivation, resulting in fetal sequelae. To investigate the possible association between maternal exposure to ToRCH pathogens during pregnancy and risk of ASD in the offspring, we examined the history of infection over the course of gestation by quantitating levels of agent-specific immunoglobulin G (IgG) in plasma collected at two points in time during pregnancy.

## RESULTS

A total of 1,781 plasma samples (903 samples acquired at midpregnancy and 878 acquired after delivery) from 442 mothers of ASD children and 464 mothers of control children were available for analysis. Characteristics of the study sample are shown in Table 1. Mothers of ASD children were younger, less likely to have college- or university-level education, and more likely to be first-time mothers; however, between-group differences were statistically significant only for maternal parity, with ASD children more likely to be born to first-time mothers (*P* = 0.01).

**TABLE 1:** Distribution of characteristics among study subjects

Characteristic	No. (%) of subjects by group		*P* value
	ASD	Control	
Total no. of mothers in the study	442	464	
Maternal characteristics			
Age, yr			0.13
<25	78 (17.6)	59 (12.7)	
25–29	138 (31.2)	138 (29.7)	
30–34	153 (34.6)	184 (39.7)	
≥35	73 (16.5)	83 (17.9)	
Education, yr			0.15
<12	51 (11.5)	44 (9.5)	
12	136 (30.8)	138 (29.7)	
13–16	143 (32.4)	156 (33.6)	
≥17	68 (15.4)	100 (21.6)	
Missing data	44 (10.0)	26 (5.6)	
Occupation			0.77
Employed/student	353 (79.9)	394 (84.9)	
Unemployed/benefits	44 (10.0)	46 (9.9)	
Missing data	45 (10.2)	24 (5.2)	
Smoking during pregnancy			0.21
No	352 (79.6)	400 (86.2)	
Yes	60 (13.6)	53 (11.4)	
Missing data	30 (6.8)	11 (2.4)	
Parity			0.01
0	236 (53.4)	208 (44.8)	
≥1	206 (46.6)	256 (55.2)	
Living status			0.75
Married/cohabiting	384 (86.9)	424 (91.4)	
Single/other	22 (5.0)	22 (4.7)	
Missing data	36 (8.1)	18 (3.9)	
Paternal characteristics			
Age, yr			0.43
<25	33 (7.5)	29 (6.3)	
25–29	110 (24.9)	97 (20.9)	
30–34	156 (35.3)	177 (38.1)	
35–39	86 (19.5)	106 (22.8)	
≥40	54 (12.2)	54 (11.6)	
Missing data	3 (0.7)	1 (0.2)	
Education, yr			0.20
<12	63 (14.3)	54 (11.6)	
12	168 (38.0)	170 (36.6)	
13–16	77 (17.4)	104 (22.4)	
≥17	67 (15.2)	82 (17.7)	
Missing data	67 (15.2)	54 (11.6)	
Occupation			0.06
Employed/student	369 (83.5)	432 (93.1)	
Unemployed/benefits	19 (4.3)	11 (2.4)	
Missing data	54 (12.2)	21 (4.5)	
Sex of child			0.16
Boy	364 (82.4)	365 (78.7)	
Girl	78 (17.6)	99 (21.3)	

The ToRCH assay results are presented in Table 2. Because rubella vaccination is part of the routine child vaccination schedule in Norway, almost all individuals had IgG antibodies to rubella virus. For boys, no significant differences were found between cases and controls for midpregnancy or postpartum maternal seropositivity to any of the pathogens.

**TABLE 2:** Proportion of mothers seropositive for *Toxoplasma gondii*, rubella virus, CMV, HSV-1, and HSV-2 at time of sample collection, stratified by sex of child

Pathogen	Sample collection time	Boys			Girls		
		ASD (*n*, %)	Control (*n*, %)	*P* value	ASD (*n*, %)	Control (*n*, %)	*P* value
HSV-1	Midpregnancy	191 (54.0)	183 (50.7)	0.38	36 (47.4)	60 (61.2)	0.07
	Postpartum	178 (52.7)	175 (48.3)	0.25	35 (47.3)	59 (61.5)	0.07
HSV-2	Midpregnancy	48 (13.3)	44 (12.2)	0.65	14 (18.4)	13 (13.5)	0.38
	Postpartum	49 (14.5)	38 (10.5)	0.11	12 (16.4)	15 (15.5)	0.86
CMV	Midpregnancy	197 (55.6)	207 (57.3)	0.65	49 (65.3)	54 (55.1)	0.17
	Postpartum	187 (55.7)	197 (56.3)	0.89	42 (60.0)	52 (54.2)	0.45
*Toxoplasma*	Midpregnancy	36 (10.0)	37 (10.2)	0.95	9 (11.7)	9 (9.3)	0.60
	Postpartum	35 (10.3)	36 (10.0)	0.89	8 (11.0)	9 (9.1)	0.69
Rubella	Midpregnancy	358 (98.9)	364 (99.7)	0.18	77 (100)	98 (99.0)	0.38
	Postpartum	337 (98.8)	363 (99.7)	0.16	74 (100)	98 (100)	NA^a^

a NA, not applicable.

Correspondingly in girls, apparent differences for CMV, HSV-1, or HSV-2 serostatus between cases and controls were not statistically significant at either the midpregnancy or postpartum time point.

The rate of seroconversion consistent with primary infection from midgestation to birth was assessed with the 875 mothers (412 cases and 463 controls) who had samples available at both time points. During pregnancy, eight women acquired antibodies to CMV, four acquired antibodies to HSV-1, four acquired antibodies to *T. gondii*, and two acquired antibodies to HSV-2. Of these, one individual changed status from seronegative to seropositive for both CMV and *T. gondii*, one changed for both CMV and HSV-1, and one changed for both HSV-1 and HSV-2.

**FIG 1:**
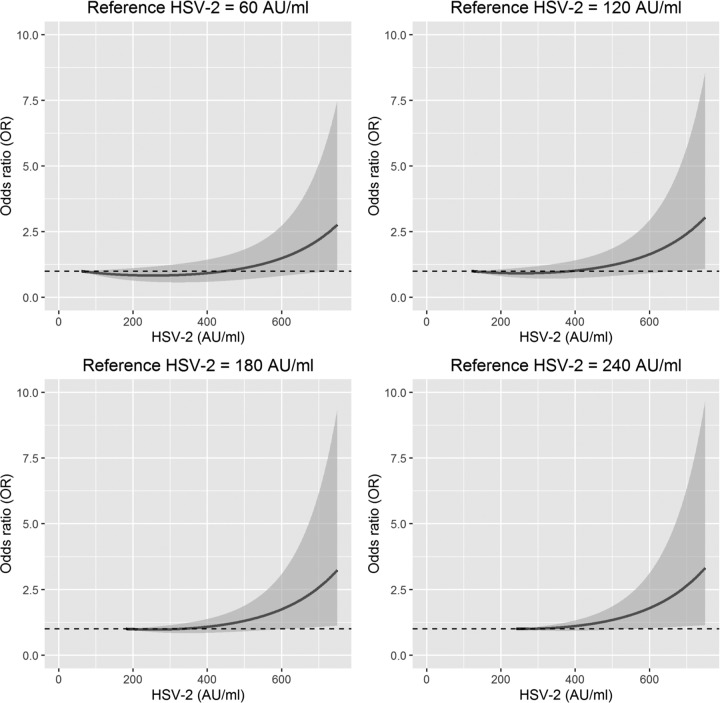
Association between maternal antibodies against herpes simplex virus 2 (HSV-2) at midpregnancy and the risk of autism spectrum disorder (ASD) in boys represented as odds ratios (ORs) with 95% confidence intervals (CIs) for four different reference levels (60, 120, 180, and 240 AU/ml) of anti-HSV-2. ORs are based on results obtained with logistic models using HSV-2 levels as linear and quadratic predictor variables and ASD as a binary response variable. Model is adjusted for parity and child’s year of birth.

The respective mean levels of antibodies measured at midpregnancy and postpartum, stratified by sex of the child, appear in Table 3. The Mann-Whitney *U* test indicated significant differences between cases and controls in levels of antibodies to HSV-2 in midpregnancy samples from mothers of boys (*U* = 59,636, *P* < 0.05, *P* [adjusted for multiple comparison] = 0.12). This trend led us to examine relationships between HSV-2 infection and ASD in boys using other statistical models. Accordingly, we estimated the strength of association by calculating the odds ratios (ORs) from a logistic model using both a linear and a quadratic term of anti-HSV-2. The quadratic term can be viewed as an interaction term in which the OR depends on the anti-HSV-2 value used as a reference. Figure 1 displays the OR with 95% confidence interval (CI) for four different anti-HSV-2 reference levels (60, 120, 180, and 240 arbitrary units [AU]/ml). Given our fitted logistic model, we calculated ORs with corresponding CIs for any additive increase in anti-HSV-2 levels relative to one of the four reference values. As examples of our approach, in the top left panel of Fig. 1, an anti-HSV-2 level of 200 AU/ml (i.e., above the 120-AU/ml cutoff for seropositivity) resulted in an OR of 0.84 (95% CI, 0.64 to 1.11; *P* value = 0.22) compared to a reference level of 60 AU/ml (seronegative subjects). As shown in the lower right panel of Fig. 1, an anti-HSV-2 level of 640 AU/ml (seropositive; high level) yielded an OR of 2.07 (95% CI, 1.06 to 4.06; *P* value = 0.03) compared to subjects with a reference level of 240 AU/ml (seropositive; low level). Estimates were adjusted for maternal parity and child’s year of birth. High levels of antibodies, which are typically indicative of recent infection, were found in only a small number of subjects, resulting in wide confidence intervals (see Fig. S1 in the supplemental material). Similar analyses revealed no statistically significant association between ASD and maternal levels of anti-HSV-2 antibodies at delivery or in samples from mothers of girls (data not shown).

10.1128/mSphere.00016-17.2 FIG S1  Number of subjects with an anti-herpes simplex virus 2 (HSV-2) titer in arbitrary units per milliliter as large as or larger than the given anti-HSV-2 value. Download FIG S1, PDF file, 0.1 MB. Copyright © 2017 Mahic et al. 2017 Mahic et al. This content is distributed under the terms of the Creative Commons Attribution 4.0 International license.

**TABLE 3:** Levels of maternal antibodies against *Toxoplasma gondii*, rubella virus, CMV, HSV-1, and HSV-2 measured in samples collected at midpregnancy and postpartum, stratified by sex of child^b^

Antibody at time	Boys			Girls		
	ASD	Controls	*P* value	ASD	Controls	*P* value
Midpregnancy (*n*)	362	365		77	99	
Anti-HSV-1	295.08 (300.40)	288.09 (301.60)	0.71	281.25 (299.40)	327.64 (306.30)	0.43
Anti-HSV-2	75.99 (173.21)^a^	62.18 (126.33)^a^	0.02	71.70 (150.47)	72.32 (154.86)	0.67
Anti-CMV	198.64 (183.45)	197.67 (184.66)	0.53	192.23 (163.14)	171.22 (153.11)	0.34
Anti-*Toxoplasma*	51.10 (102.67)	54.62 (106.92)	0.15	57.71 (108.36)	51.82 (100.70)	0.53
Anti-rubella virus	79.34 (31.77)	82.62 (29.53)	0.10	81.36 (32.87)	80.53 (33.31)	0.78
Postpartum (*n*)	341	364		74	99	
Anti-HSV-1	277.01 (288.63)	259.48 (286.56)	0.35	262.59 (293.53)	307.87 (301.07)	0.35
Anti-HSV-2	72.43 (165.31)	57.59 (130.10)	0.16	66.47 (157.84)	71.60 (155.05)	0.92
Anti-CMV	192.58 (186.73)	183.56 (173.63)	0.82	178.71 (164.66)	155.25 (141.74)	0.55
Anti-*Toxoplasma*	43.95 (87.22)	47.67 (95.71)	0.71	48.24 (79.94)	46.74 (97.47)	0.05
Anti-rubella virus	70.32 (29.75)	73.94 (29.50)	0.08	74.67 (31.20)	70.92 (32.17)	0.38

a Mann-Whitney *U* = 59,636; *P* = 0.02, two-tailed; adjusted for multiple comparison between pathogens, *P* = 0.12.

b Unless indicated otherwise, values are mean antibody levels (SD) reported as arbitrary units per milliliter, except for anti-rubella virus, which is reported as international units per milliliter.

In general, a modest decline in levels of antibodies to each of the ToRCH pathogens was observed from midpregnancy to delivery for mothers of ASD children as well as mothers of non-ASD children. The decrease was slightly lower for cases than for controls, but the association was not statistically significant for any of the pathogens (data not shown).

## DISCUSSION

This is the first study to report an association between maternal anti-HSV-2 antibody levels and risk of ASD in offspring. Our data suggest that the presence of high levels of anti-HSV-2 antibodies at midpregnancy increases the risk of ASD in boys.

Studies examining the role of gestational infection in the etiology of ASD have been primarily based on self-reported infections or in-hospital databases (23, 24). Published literature investigating the role of ToRCH agents in ASD has focused chiefly on the antibody status of individuals diagnosed with ASD rather than their mothers (25, 26). Our strategy differs in that we used plasma samples taken from mothers at midpregnancy and after delivery to test for associations between the presence of maternal antibodies to ToRCH pathogens at two points in time and risk of ASD in the offspring.

Approximately 70% of the Norwegian adult population has antibodies to HSV-1 (27); the prevalence of antibodies to CMV and HSV-2 in pregnant Norwegian women is 70% and 27%, respectively (28, 29). In our study, immunoreactivity prevalence proportion rates of 50% for HSV-1, 58% for CMV, and 15% for HSV-2 were lower for all agents except *T. gondii*, where the seroprevalence of 10% was consistent with other studies (30). Differences may reflect variations in assays used and/or characteristics of the study populations. In general, Norwegian Mother and Child Cohort Study (MoBa) participants have healthier lifestyle patterns and are more highly educated than nonparticipants (2). Overall, less than 1% of women seroconverted to any ToRCH agent during pregnancy. In general, all anti-ToRCH antibody levels were slightly lower postpartum than at midpregnancy, likely due to hemodilution but potentially also related to waning antibody responses (31–34).

The elevated antibody levels to HSV-2 may indicate either recent primary infection or reactivation of latent infection. Only 12% of HSV-2-seropositive mothers reported having HSV lesions before pregnancy or during the first trimester. HSV-2 infections can be asymptomatic; thus, we cannot rule out the possibility that some of the individuals in this study acquired primary infection just prior to, or in the first weeks of, pregnancy (35, 36).

Infection with HSV *in utero* accounts for only 4 to 9% of HSV cases diagnosed at birth. Transmission typically occurs during labor and delivery and may cause death, neurologic deficits, blindness, seizures, and learning disabilities (37, 38). In uncommon instances when HSV-2 traverses the placenta, infection results in multiorgan damage and frequently culminates in death *in utero* (39, 40). It is unlikely therefore that elevated maternal gestational antibody titers to HSV-2 reflect fetal infection.

The prevalence of autoimmune diseases is higher both in mothers of ASD children and in individuals with ASD (41–43). Autoantibodies found in some mothers of ASD children have been shown to alter behavioral outcomes in offspring in animal models (44, 45). However, to our knowledge, there are no examples of antibodies that are cross-reactive between HSV-2 and human brain. Furthermore, we are unaware of correlations between level of antibodies and autoreactivity.

A wide range of infectious agents has been linked to ASD, suggesting the possibility that general immune activation in susceptible subjects, rather than a specific pathogen *per se*, is associated with risk of ASD. Transfer of maternally produced antibodies and cytokines across the placenta and/or exposure of the fetus to inflammatory molecules produced by the placenta and decidua in relation to viral shedding may lead to fetal brain inflammation. HSV-2 shedding has been reported to occur simultaneously in distinct regions of the genital tract, independently of lesions linked to more localized inflammation (46–48), and is associated with increased levels of interleukin 6 (IL-6) and tumor necrosis factor alpha (TNF-α) (49, 50). There is evidence that these cytokines can enter fetal circulation (51–54), cross the blood-brain barrier (BBB), and stimulate production of inflammatory cytokines by microglial cells and astrocytes in fetal brain, further increasing the permeability of the BBB and damaging the developing white matter (55–57). Elevated levels of cytokines, including TNF-α, have also been found in the amniotic fluid of offspring who were later diagnosed with ASD (58).

The number of ASD females (*n* = 78) in our study is too small to conclude that the effect is sex specific; nonetheless, autism has a sex bias skewed toward males (4, 59). Trophoblasts from male placentae respond to infection-related signals with higher levels of proinflammatory cytokines (60, 61). Furthermore, male births may be prone to greater adversity, including preterm birth, in pregnancies involving infection (62, 63).

In work reported here, we found that, whereas increased risk of ASD was associated with high levels of HSV-2 antibodies at midpregnancy, no statistically significant association with risk was found with high levels of HSV-2 antibodies at delivery. Our findings are consistent with experimental data from mouse models of gestational infection wherein vulnerability of offspring to neurodevelopmental damage depends on timing of the infection of the maternal host and associated activation of the prenatal innate immune system (64). Epidemiological data suggest that activation of the maternal immune system during early to midpregnancy is associated with long-term developmental brain and behavioral abnormality in the offspring. Elevated serum levels of gamma interferon (IFN-γ), interleukin-4 (IL-4), and interleukin-5 (IL-5) in samples taken at midpregnancy from mothers have been linked to increased risk of ASD (65, 66). Recent work suggests that the association between increased maternal midpregnancy proinflammatory cytokines and ASD risk may be restricted to ASD individuals with comorbid intellectual deficiency (67). We have only limited information for intellectual quotient for the ASD subjects included in this study. Thus, we cannot rigorously investigate the relevance of our findings with regard to intellectual comorbidities.

A limitation in our study includes potential bias due to differential absence of plasma samples. The only observed pattern for the absence of samples was that all missing samples were postpartum samples in case mothers. If these samples were missing because of complications during either pregnancy or delivery, the risk would be overestimated. Restricting the analysis of midpregnancy anti-HSV-2 antibody levels to mothers with both midpregnancy and delivery samples resulted in an even stronger association between anti-HSV-2 antibody levels and risk of ASD in boys.

We speculate that ASD risk associated with high levels of antibodies to HSV-2 is not specific to HSV-2 but instead reflects the impact of immune activation and inflammation on a vulnerable developing nervous system. The replicability of these findings should be tested in other cohorts given the potential implications for serological monitoring of HSV-2 infections and opportunities for implementation of suppressive therapy during pregnancy. Broader serosurveys should also be implemented to examine whether other infectious agents have similar impacts on the incidence of neurodevelopmental disorders.

## MATERIALS AND METHODS

### Study subjects.

The Autism Birth Cohort (ABC) study is a case-control study nested within the Norwegian Mother and Child Cohort Study (MoBa), conducted by the Norwegian Institute of Public Health. The MoBa recruited pregnant women in Norway from 1999 to 2008 (68) and includes 114,479 children, 95,244 mothers, and 75,500 fathers. Maternal blood samples were collected at midpregnancy (around week 18) and after delivery, processed to extract plasma within 30 min, and stored at −80°C (68). Research for the ABC study, and specifically the research presented in this manuscript, was performed under the auspices of the Columbia University Medical Center Institutional Review Board (protocol no. AAAA2258) and the Regional Committee for Medical and Health Research Ethics for Southeastern Norway.

Children with ASD were identified through questionnaire screening of mothers at offspring ages 3, 5, and 7 years; professional and parental referrals of participants suspected of having ASD; and linkages to the Norwegian Patient Register (NPR). A subset of children was diagnosed at the ABC clinic in Oslo (69).

Our study group included 412 mothers of ASD cases with two plasma samples (obtained at midpregnancy and after delivery), 30 mothers of ASD cases with one plasma sample (obtained either at midpregnancy or after delivery), 463 mothers of non-ASD subjects (controls) with two plasma samples (obtained at midpregnancy and after delivery), and 1 mother of a non-ASD subject (control) with a sample obtained at midpregnancy. Non-ASD subject controls were frequency matched on sex, birth month, and birth year. Matching was done at an early stage of the study, resulting in small differences in numbers of samples of cases and controls at the time that laboratory analyses were performed. Multiple-gestation pregnancies were excluded (see Table S1 in the supplemental material).

10.1128/mSphere.00016-17.1 TABLE S1  Gestational age (GA) of the child at time of sample collection from the mothers in the study. Download TABLE S1, DOCX file, 0.1 MB. Copyright © 2017 Mahic et al. 2017 Mahic et al. This content is distributed under the terms of the Creative Commons Attribution 4.0 International license.

### Laboratory analyses: ToRCH.

Levels of IgG antibodies to *T. gondii*, rubella virus, CMV, HSV-1, and HSV-2 in plasma were measured using the Zeus AtheNA Multi-Lyte ToRCH IgG Plus test system (Athena; Zeus Scientific, Inc., NJ, USA). All plasma samples were run in duplicate with negative and positive controls and in-house human control plasma samples by investigators blind to the case/control status.

The raw data were analyzed using Athena software. For initial categorical interpretation of serostatus, we used the manufacturer’s cutoff values: <100 arbitrary units (AU)/ml for negative, between 100 and 120 AU/ml for equivocal, and >120 AU/ml for positive for *T. gondii*, CMV, HSV-1, and HSV-2. For rubella virus, we employed the manufacturer’s cutoffs of <10 IU/ml for negative, 10 IU/ml for equivocal, and >10 IU/ml for positive.

### Covariates.

Variables potentially influencing both the risk of being seropositive for any of the pathogens and the risk of ASD were identified as possible confounders. The following covariates were tested for confounding: maternal age at delivery, maternal smoking during pregnancy, parity, and maternal education.

### Statistical analysis.

Data were analyzed using IBM SPSS Statistics for Windows, version 23.0 (IBM Corp., Armonk, NY, 2015), MatLab and Statistics Toolbox release 2013a (The MathWorks, Inc., Natick, MA), and RStudio running R version 3.3.1 (RStudio, Inc., Boston, MA). All analyses were done for mothers of girls and boys separately. Characteristics of controls and cases were compared using chi-square tests, and all *P* values are two-tailed. Chi-square tests were also used to test whether the number of seropositive individuals differed between case and control groups. Adjustments for multiple comparisons between pathogens were performed using the false discovery rate (FDR) method at an 0.05 level of significance. We applied binary logistic regression to estimate crude and adjusted odds ratios (ORs) of ASD in the offspring, with associated 95% confidence intervals (CIs). Both linear and quadratic terms of antibody levels were included in order to model a nonlinear relation between immunoreactivity and log odds of ASD. Analysis of antibody levels by fitting a logistic regression model with linear and quadratic terms gave the best fit based on Bayesian information criterion. Since the quadratic term can be viewed as an interaction term in which the OR depends on the antibody levels used as a reference, different reference levels were used in order to display the results for a single fitted model. Explicitly, given a single fitted logistic model, we calculated ORs with corresponding CIs for any additive increase in antibody levels relative to the set of reference values.

Repeated measures of antibody levels were modeled using the generalized estimating equations model (GEE). Case-control status was entered as the between-group variable, together with week of gestation at sampling as the within-subject variable. Potential interactions between case-control status and week of gestation (both midgestational and birth) were tested. A statistical significance level of 0.05 was used for all analyses.

## References

[1] American Psychiatric Association 2013 Diagnostic and statistical manual of mental disorders, 5th ed. American Psychiatric Association, Washington, DC.

[2] Ozonoff S, Goodlin-Jones BL, Solomon M 2005 Evidence-based assessment of autism spectrum disorders in children and adolescents. J Clin Child Adolesc Psychol 34:523–540. doi:10.1207/s15374424jccp3403_8. 16083393

[3] Elsabbagh M, Divan G, Koh YJ, Kim YS, Kauchali S, Marcín C, Montiel-Nava C, Patel V, Paula CS, Wang C, Yasamy MT, Fombonne E 2012 Global prevalence of autism and other pervasive developmental disorders. Autism Res 5:160–179. doi:10.1002/aur.239. 22495912 PMC3763210

[4] Werling DM, Geschwind DH 2013 Sex differences in autism spectrum disorders. Curr Opin Neurol 26:146–153. doi:10.1097/WCO.0b013e32835ee548. 23406909 PMC4164392

[5] Hallmayer J, Cleveland S, Torres A, Phillips J, Cohen B, Torigoe T, Miller J, Fedele A, Collins J, Smith K, Lotspeich L, Croen LA, Ozonoff S, Lajonchere C, Grether JK, Risch N 2011 Genetic heritability and shared environmental factors among twin pairs with autism. Arch Gen Psychiatry 68:1095–1102. doi:10.1001/archgenpsychiatry.2011.76. 21727249 PMC4440679

[6] Lyall K, Schmidt RJ, Hertz-Picciotto I 2014 Maternal lifestyle and environmental risk factors for autism spectrum disorders. Int J Epidemiol 43:443–464. doi:10.1093/ije/dyt282. 24518932 PMC3997376

[7] Brown AS 2012 Epidemiologic studies of exposure to prenatal infection and risk of schizophrenia and autism. Dev Neurobiol 72:1272–1276. doi:10.1002/dneu.22024. 22488761 PMC3435457

[8] Buka SL, Cannon TD, Torrey EF, Yolken RH, Collaborative Study Group on the Perinatal Origins of Severe Psychiatric Disorders 2008 Maternal exposure to herpes simplex virus and risk of psychosis among adult offspring. Biol Psychiatry 63:809–815. doi:10.1016/j.biopsych.2007.09.022. 17981263

[9] Matelski L, Van de Water J 2016 Risk factors in autism: thinking outside the brain. J Autoimmun 67:1–7. doi:10.1016/j.jaut.2015.11.003. 26725748 PMC5467975

[10] Patterson PH 2002 Maternal infection: window on neuroimmune interactions in fetal brain development and mental illness. Curr Opin Neurobiol 12:115–118. 11861174 10.1016/s0959-4388(02)00299-4

[11] Estes ML, McAllister AK 2015 Immune mediators in the brain and peripheral tissues in autism spectrum disorder. Nat Rev Neurosci 16:469–486. doi:10.1038/nrn3978. 26189694 PMC5650494

[12] Hornig M 2013 The role of microbes and autoimmunity in the pathogenesis of neuropsychiatric illness. Curr Opin Rheumatol 25:488–795. doi:10.1097/BOR.0b013e32836208de. 23656715

[13] De Miranda J, Yaddanapudi K, Hornig M, Villar G, Serge R, Lipkin WI 2010 Induction of toll-like receptor 3-mediated immunity during gestation inhibits cortical neurogenesis and causes behavioral disturbances. mBio 1:e00176-10. doi:10.1128/mBio.00176-10. 20941330 PMC2953007

[14] Malkova NV, Yu CZ, Hsiao EY, Moore MJ, Patterson PH 2012 Maternal immune activation yields offspring displaying mouse versions of the three core symptoms of autism. Brain Behav Immun 26:607–616. doi:10.1016/j.bbi.2012.01.011. 22310922 PMC3322300

[15] Shet A 2011 Congenital and perinatal infections: throwing new light with an old TORCH. Indian J Pediatr 78:88–95. doi:10.1007/s12098-010-0254-3. 20953849

[16] Neu N, Duchon J, Zachariah P 2015 TORCH infections. Clin Perinatol 42:77–103. doi:10.1016/j.clp.2014.11.001. 25677998

[17] Chemla C, Villena I, Aubert D, Hornoy P, Dupouy D, Leroux B, Bory JP, Pinon JM 2002 Preconception seroconversion and maternal seronegativity at delivery do not rule out the risk of congenital toxoplasmosis. Clin Diagn Lab Immunol 9:489–490. doi:10.1128/CDLI.9.2.489-490.2002. 11874899 PMC119954

[18] Dollfus H, Dureau P, Hennequin C, Uteza Y, Bron A, Dufier JL 1998 Congenital toxoplasma chorioretinitis transmitted by preconceptionally immune women. Br J Ophthalmol 82:1444–1445. 9930281 10.1136/bjo.82.12.1444 PMC1722451

[19] Robert-Gangneux F, Yera H, D’Herve D, Guiguen C 2009 Congenital toxoplasmosis after a preconceptional or periconceptional maternal infection. Pediatr Infect Dis J 28:660–661. doi:10.1097/INF.0b013e3181966020. 19561432

[20] Vogel N, Kirisits M, Michael E, Bach H, Hostetter M, Boyer K, Simpson R, Holfels E, Hopkins J, Mack D, Mets MB, Swisher CN, Patel D, Roizen N, Stein L, Stein M, Withers S, Mui E, Egwuagu C, Remington J, Dorfman R, McLeod R 1996 Congenital toxoplasmosis transmitted from an immunologically competent mother infected before conception. Clin Infect Dis 23:1055–1060. 8922802 10.1093/clinids/23.5.1055

[21] Landais I, Nelson JA 2013 Functional genomics approaches to understand cytomegalovirus replication, latency and pathogenesis. Curr Opin Virol 3:408–415. doi:10.1016/j.coviro.2013.06.002. 23816389 PMC3748260

[22] Roizman B, Whitley RJ 2013 An inquiry into the molecular basis of HSV latency and reactivation. Annu Rev Microbiol 67:355–374. doi:10.1146/annurev-micro-092412-155654. 24024635

[23] Atladóttir HÓ, Henriksen TB, Schendel DE, Parner ET 2012 Autism after infection, febrile episodes, and antibiotic use during pregnancy: an exploratory study. Pediatrics 130:e1447–e1454. doi:10.1542/peds.2012-1107. 23147969 PMC4451062

[24] Atladóttir HO, Thorsen P, Østergaard L, Schendel DE, Lemcke S, Abdallah M, Parner ET 2010 Maternal infection requiring hospitalization during pregnancy and autism spectrum disorders. J Autism Dev Disord 40:1423–1430. doi:10.1007/s10803-010-1006-y. 20414802

[25] Gentile I, Zappulo E, Bonavolta R, Maresca R, Messana T, Buonomo AR, Portella G, Sorrentino R, Settimi A, Pascotto A, Borgia G, Bravaccio C 2014 Prevalence and titre of antibodies to cytomegalovirus and Epstein-Barr virus in patients with autism spectrum disorder. In Vivo 28:621–626. 24982232

[26] Gentile I, Zappulo E, Bonavolta R, Maresca R, Riccio MP, Buonomo AR, Portella G, Vallefuoco L, Settimi A, Pascotto A, Borgia G, Bravaccio C 2014 Prevalence of herpes simplex virus 1 and 2 antibodies in patients with autism spectrum disorders. In Vivo 28:667–671. 24982239

[27] Olsen AO, Orstavik I, Dillner J, Vestergaard BF, Magnus P 1998 Herpes simplex virus and human papillomavirus in a population-based case-control study of cervical intraepithelial neoplasia grade II-III. APMIS 106:417–424. 9548432 10.1111/j.1699-0463.1998.tb01366.x

[28] Eskild A, Jeansson S, Stray-Pedersen B, Jenum PA 2002 Herpes simplex virus type-2 infection in pregnancy: no risk of fetal death: results from a nested case-control study within 35,940 women. BJOG 109:1030–1035. 12269678 10.1111/j.1471-0528.2002.01534.x

[29] Eskild A, Jenum PA, Bruu AL 2005 Maternal antibodies against cytomegalovirus in pregnancy and the risk of fetal death and low birth weight. Acta Obstet Gynecol Scand 84:1035–1041. doi:10.1111/j.0001-6349.2005.00796.x. 16232169

[30] Jenum PA, Stray-Pedersen B, Melby KK, Kapperud G, Whitelaw A, Eskild A, Eng J 1998 Incidence of Toxoplasma gondii infection in 35,940 pregnant women in Norway and pregnancy outcome for infected women. J Clin Microbiol 36:2900–2906. 9738041 10.1128/jcm.36.10.2900-2906.1998 PMC105085

[31] Yasuhara M, Tamaki H, Iyama S, Yamaguchi Y, Tachi J, Amino N 1992 Reciprocal changes in serum levels of immunoglobulins (IgG, IgA, IgM) and complements (C3, C4) in normal pregnancy and after delivery. J Clin Lab Immunol 38:137–141. 1345750

[32] Amino N, Tanizawa O, Miyai K, Tanaka F, Hayashi C, Kawashima M, Ichihara K 1978 Changes of serum immunoglobulins IgG, IgA, IgM, and IgE during pregnancy. Obstet Gynecol 52:415–420. 714321

[33] Miller EC, Abel W 1984 Changes in the immunoglobulins IgG, IgA and IgM in pregnancy and the puerperium. Zentralbl Gynakol 106:1084–1091. (In German.) 6495924

[34] Abel J, Conklin J, Hunter S, Empey R, Tyler E, Christensen A, Talcott K, Ballas Z, Santillan M, Santillan D 2013 Defining normal IgG changes throughout pregnancy. Proc Obstet Gynecol 3:

[35] Enright AM, Prober CG 2004 Herpesviridae infections in newborns: varicella zoster virus, herpes simplex virus, and cytomegalovirus. Pediatr Clin North Am 51:889–908. doi:10.1016/j.pcl.2004.03.005. 15275980

[36] Sacks SL, Griffiths PD, Corey L, Cohen C, Cunningham A, Dusheiko GM, Self S, Spruance S, Stanberry LR, Wald A, Whitley RJ 2004 HSV shedding. Antiviral Res 63(Suppl 1):S19–S26. doi:10.1016/j.antiviral.2004.06.004. 15450382

[37] Brown ZA, Wald A, Morrow RA, Selke S, Zeh J, Corey L 2003 Effect of serologic status and cesarean delivery on transmission rates of herpes simplex virus from mother to infant. JAMA 289:203–209. doi:10.1001/jama.289.2.203. 12517231

[38] Thompson C, Whitley R 2011 Neonatal herpes simplex virus infections: where are we now? Adv Exp Med Biol 697:221–230. doi:10.1007/978-1-4419-7185-2_15. 21120729 PMC3433171

[39] Avgil M, Ornoy A 2006 Herpes simplex virus and Epstein-Barr virus infections in pregnancy: consequences of neonatal or intrauterine infection. Reprod Toxicol 21:436–445. doi:10.1016/j.reprotox.2004.11.014. 16580943

[40] Whitley RJ, Kimberlin DW, Roizman B 1998 Herpes simplex viruses. Clin Infect Dis 26:541–553. 9524821 10.1086/514600

[41] Atladóttir HO, Pedersen MG, Thorsen P, Mortensen PB, Deleuran B, Eaton WW, Parner ET 2009 Association of family history of autoimmune diseases and autism spectrum disorders. Pediatrics 124:687–694. doi:10.1542/peds.2008-2445. 19581261

[42] Croen LA, Grether JK, Yoshida CK, Odouli R, Van de Water J 2005 Maternal autoimmune diseases, asthma and allergies, and childhood autism spectrum disorders: a case-control study. Arch Pediatr Adolesc Med 159:151–157. doi:10.1001/archpedi.159.2.151. 15699309

[43] Kohane IS, McMurry A, Weber G, MacFadden D, Rappaport L, Kunkel L, Bickel J, Wattanasin N, Spence S, Murphy S, Churchill S 2012 The co-morbidity burden of children and young adults with autism spectrum disorders. PLoS One 7:e33224. doi:10.1371/journal.pone.0033224. 22511918 PMC3325235

[44] Brimberg L, Sadiq A, Gregersen PK, Diamond B 2013 Brain-reactive IgG correlates with autoimmunity in mothers of a child with an autism spectrum disorder. Mol Psychiatry 18:1171–1177. doi:10.1038/mp.2013.101. 23958959

[45] Martin LA, Ashwood P, Braunschweig D, Cabanlit M, Van de Water J, Amaral DG 2008 Stereotypies and hyperactivity in rhesus monkeys exposed to IgG from mothers of children with autism. Brain Behav Immun 22:806–816. doi:10.1016/j.bbi.2007.12.007. 18262386 PMC3779644

[46] Johnston C, Corey L 2016 Current concepts for genital herpes simplex virus infection: diagnostics and pathogenesis of genital tract shedding. Clin Microbiol Rev 29:149–161. doi:10.1128/CMR.00043-15. 26561565 PMC4771215

[47] Johnston C, Zhu J, Jing L, Laing KJ, McClurkan CM, Klock A, Diem K, Jin L, Stanaway J, Tronstein E, Kwok WW, Huang ML, Selke S, Fong Y, Magaret A, Koelle DM, Wald A, Corey L 2014 Virologic and immunologic evidence of multifocal genital herpes simplex virus 2 infection. J Virol 88:4921–4931. doi:10.1128/JVI.03285-13. 24554666 PMC3993786

[48] Wald A, Zeh J, Selke S, Warren T, Ryncarz AJ, Ashley R, Krieger JN, Corey L 2000 Reactivation of genital herpes simplex virus type 2 infection in asymptomatic seropositive persons. N Engl J Med 342:844–850. doi:10.1056/NEJM200003233421203. 10727588

[49] Fernandez S, Gillgrass A, Kaushic C 2007 Differential responses of murine vaginal and uterine epithelial cells prior to and following herpes simplex virus type 2 (HSV-2) infection. Am J Reprod Immunol 57:367–377. doi:10.1111/j.1600-0897.2007.00482.x. 17430501

[50] Nason MC, Patel EU, Kirkpatrick AR, Prodger JL, Shahabi K, Tobian AA, Gianella S, Kalibbala S, Ssebbowa P, Kaul R, Gray RH, Quinn TC, Serwadda D, Reynolds SJ, Redd AD 2016 Immunological signaling during herpes simplex virus-2 and cytomegalovirus vaginal shedding after initiation of antiretroviral treatment. Open Forum Infect Dis 3:ofw073. doi:10.1093/ofid/ofw073. 27191006 PMC4867667

[51] Chow SS, Craig ME, Jones CA, Hall B, Catteau J, Lloyd AR, Rawlinson WD 2008 Differences in amniotic fluid and maternal serum cytokine levels in early midtrimester women without evidence of infection. Cytokine 44:78–84. doi:10.1016/j.cyto.2008.06.009. 18703348

[52] Dammann O, Leviton A 1997 Maternal intrauterine infection, cytokines, and brain damage in the preterm newborn. Pediatr Res 42:1–8. doi:10.1203/00006450-199707000-00001. 9212029

[53] Potter JA, Garg M, Girard S, Abrahams VM 2015 Viral single stranded RNA induces a trophoblast pro-inflammatory and antiviral response in a TLR8-dependent and -independent manner. Biol Reprod 92:17. doi:10.1095/biolreprod.114.124032. 25429091 PMC4434931

[54] Zaretsky MV, Alexander JM, Byrd W, Bawdon RE 2004 Transfer of inflammatory cytokines across the placenta. Obstet Gynecol 103:546–550. doi:10.1097/01.AOG.0000114980.40445.83. 14990420

[55] Hsiao EY, Patterson PH 2011 Activation of the maternal immune system induces endocrine changes in the placenta via IL-6. Brain Behav Immun 25:604–615. doi:10.1016/j.bbi.2010.12.017. 21195166 PMC3081363

[56] Malaeb S, Dammann O 2009 Fetal inflammatory response and brain injury in the preterm newborn. J Child Neurol 24:1119–1126. doi:10.1177/0883073809338066. 19605775 PMC3695470

[57] McAdams RM, Juul SE 2012 The role of cytokines and inflammatory cells in perinatal brain injury. Neurol Res Int 2012:561494. doi:10.1155/2012/561494. 22530124 PMC3317045

[58] Abdallah MW, Larsen N, Grove J, Nørgaard-Pedersen B, Thorsen P, Mortensen EL, Hougaard DM 2013 Amniotic fluid inflammatory cytokines: potential markers of immunologic dysfunction in autism spectrum disorders. World J Biol Psychiatry 14:528–538. doi:10.3109/15622975.2011.639803. 22175527

[59] Steeb H, Ramsey JM, Guest PC, Stocki P, Cooper JD, Rahmoune H, Ingudomnukul E, Auyeung B, Ruta L, Baron-Cohen S, Bahn S 2014 Serum proteomic analysis identifies sex-specific differences in lipid metabolism and inflammation profiles in adults diagnosed with Asperger syndrome. Mol Autism 5:4. doi:10.1186/2040-2392-5-4. 24467795 PMC3905921

[60] Challis J, Newnham J, Petraglia F, Yeganegi M, Bocking A 2013 Fetal sex and preterm birth. Placenta 34:95–99. doi:10.1016/j.placenta.2012.11.007. 23261268

[61] Yeganegi M, Watson CS, Martins A, Kim SO, Reid G, Challis JR, Bocking AD 2009 Effect of Lactobacillus rhamnosus GR-1 supernatant and fetal sex on lipopolysaccharide-induced cytokine and prostaglandin-regulating enzymes in human placental trophoblast cells: implications for treatment of bacterial vaginosis and prevention of preterm labor. Am J Obstet Gynecol 200:532.e1–532.e8. doi:10.1016/j.ajog.2008.12.032. 19285652

[62] Brettell R, Yeh PS, Impey LW 2008 Examination of the association between male gender and preterm delivery. Eur J Obstet Gynecol Reprod Biol 141:123–126. doi:10.1016/j.ejogrb.2008.07.030. 18783867

[63] McGregor JA, Leff M, Orleans M, Baron A 1992 Fetal gender differences in preterm birth: findings in a North American cohort. Am J Perinatol 9:43–48. doi:10.1055/s-2007-994668. 1550632

[64] Meyer U, Nyffeler M, Engler A, Urwyler A, Schedlowski M, Knuesel I, Yee BK, Feldon J 2006 The time of prenatal immune challenge determines the specificity of inflammation-mediated brain and behavioral pathology. J Neurosci 26:4752–4762. doi:10.1523/JNEUROSCI.0099-06.2006. 16672647 PMC6674174

[65] Goines PE, Croen LA, Braunschweig D, Yoshida CK, Grether J, Hansen R, Kharrazi M, Ashwood P, Van de Water J 2011 Increased midgestational IFN-gamma, IL-4 and IL-5 in women bearing a child with autism: a case-control study. Mol Autism 2:13. doi:10.1186/2040-2392-2-13. 21810230 PMC3170586

[66] Patterson PH 2011 Maternal infection and immune involvement in autism. Trends Mol Med 17:389–394. doi:10.1016/j.molmed.2011.03.001. 21482187 PMC3135697

[67] Jones KL, Croen LA, Yoshida CK, Heuer L, Hansen R, Zerbo O, DeLorenze GN, Kharrazi M, Yolken R, Ashwood P, Van de Water J 2017 Autism with intellectual disability is associated with increased levels of maternal cytokines and chemokines during gestation. Mol Psychiatry 22:273–279. doi:10.1038/mp.2016.77. 27217154 PMC5122473

[68] Magnus P, Birke C, Vejrup K, Haugan A, Alsaker E, Daltveit AK, Handal M, Haugen M, Høiseth G, Knudsen GP, Paltiel L, Schreuder P, Tambs K, Vold L, Stoltenberg C 2016 Cohort profile update: the Norwegian Mother and Child Cohort Study (MoBa). Int J Epidemiol 45:382–388. doi:10.1093/ije/dyw029. 27063603

[69] Stoltenberg C, Schjølberg S, Bresnahan M, Hornig M, Hirtz D, Dahl C, Lie KK, Reichborn-Kjennerud T, Schreuder P, Alsaker E, Øyen AS, Magnus P, Surén P, Susser E, Lipkin WI, ABC Study Group 2010 The Autism Birth Cohort: a paradigm for gene-environment-timing research. Mol Psychiatry 15:676–680. doi:10.1038/mp.2009.143. 20571529 PMC2892398

