# Correction for Mahic et al., “Maternal Immunoreactivity to Herpes Simplex Virus 2 and Risk of Autism Spectrum Disorder in Male Offspring”

**DOI:** 10.1128/mSphere.00154-17

**Published:** 2017-04-12

**Authors:** Milada Mahic, Siri Mjaaland, Hege Marie Bøvelstad, Nina Gunnes, Ezra Susser, Michaeline Bresnahan, Anne-Siri Øyen, Bruce Levin, Xiaoyu Che, Deborah Hirtz, Ted Reichborn-Kjennerud, Synnve Schjølberg, Christine Roth, Per Magnus, Camilla Stoltenberg, Pål Surén, Mady Hornig, W. Ian Lipkin

**Affiliations:** a Center for Infection and Immunity, Mailman School of Public Health, Columbia University, New York, New York, USA; b Norwegian Institute of Public Health, Oslo, Norway; c Department of Epidemiology, Mailman School of Public Health, Columbia University, New York, New York, USA; d New York State Psychiatric Institute, Columbia University, New York, New York, USA; e Department of Biostatistics, Mailman School of Public Health, Columbia University, New York, New York, USA; f National Institute of Neurological Disorders and Stroke, National Institutes of Health, Bethesda, Maryland, USA; g Institute of Clinical Medicine, University of Oslo, Oslo, Norway; h Nic Waals Institute, Lovisenberg Hospital, Oslo, Norway; i Department of Global Public Health and Primary Care, University of Bergen, Bergen, Norway

## AUTHOR CORRECTION

Volume 2, no. 1, e00016-17, 2017, https://doi.org/10.1128/mSphere.00016-17. The following footnote should be included in the article: M.H. and W.I.L. contributed equally to this article.

