# Supplementary figures and images for: Maternal Immunoreactivity to Herpes Simplex Virus 2 and Risk of Autism Spectrum Disorder in Male Offspring
Source: mSphere. 2017 Feb 22;2(1):e00016-17. doi: 10.1128/mSphere.00016-17 (PMC5322345; doi:10.1128/mSphere.00016-17)

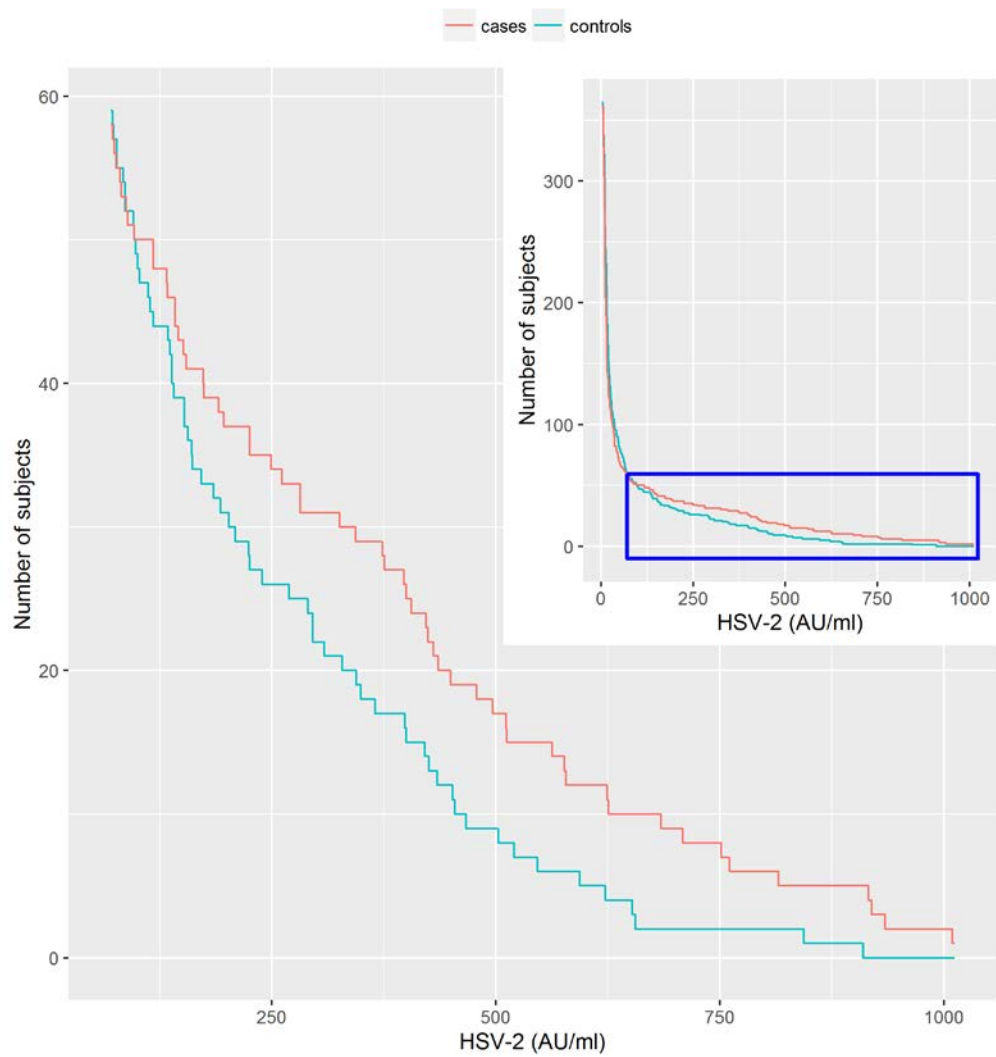

Supplement: FIG S1 [file sph001172238sf2.pdf]
