# Supplementary material for: Maternal Immunoreactivity to Herpes Simplex Virus 2 and Risk of Autism Spectrum Disorder in Male Offspring
Source: mSphere. 2017 Feb 22;2(1):e00016-17. doi: 10.1128/mSphere.00016-17 (PMC5322345; doi:10.1128/mSphere.00016-17)
Supplement: TABLE S1 [file sph001172238st1.docx]

| **Supplementary Table 1.** Gestational age (GA) of the child at time of sample collection from the mothers in the study. | | |
| --- | --- | --- |
|  |  |  |
|  | **ASD** | **controls** |
| **Total number of samples** | 854 | 927 |
| **Gestational age at sample collection, n, mean, SD** |  |  |
| Mid-pregnancy (14-39 weeks) | 439 (18.29, 1.64) | 464 (18.22, 1.41) |
| Postpartum (27-43 weeks) | 415 (39.34, 2.22) | 463 (39.58, 1.64) |
| **Mothers with samples from two timepoints** |  |  |
| Mid-pregnancy (14-39 weeks), mean, SD | 412 (18.30, 1.67) * | 463 (18.22, 1.41) |
| Postpartum (27-43 weeks), mean, SD | 412 (39.34, 2.22) * | 463 (39.58, 1.64) |
| **Mothers with only mid-pregnancy sample** |  |  |
| Mid-pregnancy (14-39 weeks), mean, SD | 27 (18.11, 1.01) | 1 (18, - ) |
| GA at birth (27-43 weeks), mean, SD | 27 (39.15, 1.70) | 1 (40, - ) |
| **Mothers with only postpartum sample** |  |  |
| Postpartum (27-43 weeks), mean, SD | 3 (40, 1.00) | 0 ( - , - ) |
| ASD: Autism Spectrum Disorder |  |  |
